# A Retrospective Analysis of the Clinicopathological Spectrum and Survival Outcome of Non-Hodgkin Lymphoma From a Tertiary Care Hospital in Saudi Arabia

**DOI:** 10.7759/cureus.97251

**Published:** 2025-11-19

**Authors:** Nosheen Mahmood, Saima Aamir, Abdullah Alseraye, Abdullah Alammari, Abdulrahman Alyahya, Khalid Alammari, Saud Alahmari

**Affiliations:** 1 Pathology, King Saud Bin Abdulaziz University for Health Sciences, Riyadh, SAU; 2 College of Medicine, King Saud Bin Abdulaziz University for Health Sciences, Riyadh, SAU

**Keywords:** albumin, biomarker, non-hodgkin lymphoma, prognostic factors, saudi arabia, survival analysis

## Abstract

Background: Non-Hodgkin lymphoma (NHL) is one of the most common malignancies worldwide. Although the incidence rate is relatively low in Saudi Arabia, the disproportionately high mortality rate highlights the need for local studies addressing the disease characteristics and prognostic factors

Methods: This was a retrospective study conducted at a tertiary care hospital in Riyadh, Saudi Arabia. Data from 252 patients diagnosed with NHL between 2017 and 2022 were reviewed and collected from electronic medical records. The variables included patients’ demographics and clinical characteristics. IBM SPSS Statistics for Windows, version 25 (IBM Corp., Armonk, New York, United States) was used for data entry and analysis. Survival outcomes were analysed using Kaplan-Meier curves, and differences between groups were evaluated using log-rank tests. A p-value of ≤ 0.05 was considered statistically significant.

Results: Diffuse large B-cell lymphoma (DLBCL) accounted for 179 (71%) cases, making it the most common type, followed by follicular NHL (FNHL), which accounted for 34 (13.5%). Almost half of the patients (n=123, 48.8%) presented at stage IV, which was associated with poor survival (p<0.0001). Bone marrow involvement was found in 75 (28.6%) patients and was associated with shorter survival (p=0.002). Patients who did not survive had a low albumin (32.9 ±6.3 vs 37.6 ±9.7) and increased lactate dehydrogenase (547± 34 vs 227±10) compared to those who were alive. Although younger age groups had longer survival than older patients, the differences did not achieve statistical significance.

Conclusions: This study provides valuable data on NHL patterns and prognosis, highlighting the critical role of early detection and the value of simple laboratory markers in predicting prognosis and improving patient outcomes.

## Introduction

Non-Hodgkin’s lymphoma (NHL) is the most frequently occurring haematological malignancy across the world. Based on the 2022 Global Cancer Observatory (GLOBOCAN) database by the International Agency for Research on Cancer (IARC), the estimated incidence of NHL was 553,389 cases worldwide, making it the 10th most common cancer globally [1]. Incidence is reported to be high in developed countries like Australia, New Zealand, North America, and Northern and Western Europe. Highest mortality has been reported in Africa, Western Asia, and Australia [2].

NHL has been ranked as the fourth most common malignancy in Saudi Arabia according to the cancer incidence report published by the Saudi Cancer Registry in 2022 [3]. It was observed to be the third most frequently diagnosed cancer in the Saudi male population, while it ranked fifth among the female population. Altowairqi et al. reported an increase in the number of NHL cases from 7% in 2009 to 11% in 2018 [4]. The Aseer region has witnessed a surge in NHL among elderly males [5].

Many variants come under the umbrella of NHL, ranging from indolent to highly aggressive subtypes. Around 90% of NHLs have a B-cell origin, only 10% originate from T cells, and less than 1% have a natural killer cell origin. Most commonly observed subtypes of B-cell NHL globally are diffuse large B-cell lymphoma (DLBCL), followed by follicular lymphoma [6]. Likewise, in Saudi Arabia, 83% of all NHLs are B-cell, and the remaining are T-cell lymphoma. Among B-cell lymphomas, the most frequently diagnosed variants are DLBCL (58%) and follicular lymphoma (9%). The majority of cases (50.4%) present at an advanced stage [7]. DLBCL accounted for 74.3% cases of NHL in a recent report based on data collected from the Al Ahsa region [8]. A higher positivity for BCL2 and BCL6 was seen in DLBCL, and the majority had an aggressive course with higher mortality.

Smoking, alcohol intake, obesity, and an unhealthy diet have been proposed as risk factors. In addition, infections, immune dysregulations, and genetic alterations have been linked to NHL development. A study conducted in Saudi Arabia aimed to assess the development of lymphomas in people with acquired immunodeficiency syndrome (AIDS) found that 5.6% developed NHL [9,10].

The International Prognostic Index classifies NHL in four risk groups, including low risk, low intermediate, high intermediate, and high risk. These categories are determined on the basis of variables including age above 60, stage III and IV, extra lymphatic involvement, poor performance status of the patient, and high lactate dehydrogenase level (LDH). Subjects with a score of 0 or 1 fall in low-risk, 2 low-intermediate, 3 high-intermediate, and 4 or 5 in high-risk groups [11].

NHL is one of the leading cancers in Saudi Arabia, and it is thus very important to study and analyze its patterns and prognostic factors. The scarcity of available data from Saudi Arabia and the diversity of reported patterns are further reasons why such a study is needed. The objectives of this study were to delineate the histopathological and clinical patterns of NHL in a tertiary care hospital in Saudi Arabia. Additionally, to determine the distribution of NHL subtypes and to document the impact of demographic and biochemical parameters on prognosis and overall survival in NHL. This study will provide valuable insights into sociodemographic and prognostic factors of lymphoma, helping health care professionals to design and streamline management guidelines for NHL in Saudi Arabia.

## Materials and methods

This was a retrospective study of patients diagnosed with NHL at King Abdulaziz Medical City in Riyadh, Saudi Arabia, between 2017 and 2022. The study was approved by the Institutional Review Board, King Abdullah International Medical Research Center (protocol number: SP23R/095/05). The requirement for patient consent was waived owing to the study design.

Eligibility criteria

The study included adult patients between 20-80 years, with a histologically confirmed diagnosis of NHL based on tissue biopsy, visiting the Haematology/Oncology department within the study period (2017-2022), were included. Patients with severe comorbidities that interfered with follow-up or impacted prognosis, irrespective of NHL, were excluded from the study. Any prior history of haematological malignancy was considered an exclusion criterion.

Sample size

A sample of 264 participants was calculated using the Raosoft online sample size calculator (http://www.raosoft.com/samplesize.html). The margin of error was estimated to be 6% based on a 95% confidence level.

Data collection

Data were collected from the electronic medical records of patients diagnosed with NHL using a predesigned data collection sheet on various variables, including age, gender, presence of B symptoms, and bone marrow involvement, date of last follow up and survival status (alive/censored or dead). Additionally, data was also recorded for LDH, serum albumin, hemoglobin, lymph node biopsy, NHL subtype, stage, and immunohistochemical profile of the tumor. NHL subtypes were assigned using the International Classification of Diseases (ICD-10 C82-86) [12]. Stages were assigned based on the number and extent of lymph node involvement, extra lymphatic spread, and involvement of liver, lung, bone marrow, or CSF in accordance with Ann Arbor classification [13].

The main outcome variable for this study was time to death, and predictor variables included age, gender, stage, bone marrow involvement, and biochemical parameter,s including serum albumin, LDH, and haemoglobin.

Data analysis

IBM SPSS Statistics for Windows, Version 25 (Released 2017; IBM Corp., Armonk, New York, United States) was used for data entry and analysis. Quantitative variables like age, LDH, serum albumin, and haemoglobin were presented as mean±standard deviation (SD). Qualitative variables like gender, stage, histological type, immunohistochemical profile, and bone marrow involvement were presented as frequencies and percentages. To compare qualitative variables between groups, the Chi-square test was applied. For the comparison of quantitative variables, Student's t-test was used for two groups and one-way analysis of variance (ANOVA) followed by a post hoc test for more than two groups.

Survival estimates were calculated for the time period 2017-2022. Univariate analysis was carried out using the Kaplan-Meier survival analysis function. Survival curves were generated to compare the groups, and Log-rank test was applied to check the significance. For multivariate analysis Cox proportional hazard model was applied, and hazard risk was calculated to analyze associations. Statistical significance was set at P<0.05

## Results

A total of 252 patients with NHL were reported over the study period. The mean age at diagnosis was 53.9 ±19.4 years. There were 134 (53%) male and 118 (46.8%) female patients. Most of the patients (n=197, 78.2%) were free from B symptoms at the time of diagnosis. The majority of the patients presented with stage IV (n=123, 48.8%). While stage I and II accounted for 46 (18.3%) and 47 (18.7%) cases, respectively, stage III had the least number of cases (n=36, 14.3%), as shown in Table 1.

**Table 1:** Clinical and demographic characteristics

Characteristics		Frequency (Percentage)
Age groups	20-34	49 (19.4)
	35-54	76 (30.2)
	>55	127 (50.4)
Gender	Male	134 (53.2)
	Female	118 (46.8)
B Symptoms	Present	55 (21.8)
	Absent	197 (78.2)
Stage	I	46 (18.3)
	II	47 (18.7)
	III	36 (14.3)
	IV	123 (48.8)
Status on last follow-up	Alive	197 (78.2)
	Dead	55 (21.8)

Bone marrow involvement was documented in 75 (29.8%) patients. Most patients were diagnosed with DLBCL (n=179, 71%), followed by follicular lymphoma (n=34, 13.5%), T cell NHL (n=15, 6%), marginal zone lymphoma (MZL) (n=10, 4%), Burkitt lymphoma (n=6, 2.4%), small lymphocytic lymphoma (SLL) (n=5, 2%), and mantle cell lymphoma (n=3, 1.2%). Frequency and age distribution among different subtypes of NHL are shown in Table 2. Patients who did not survive had low hemoglobin, albumin, and raised LDH (Table 3).

**Table 2:** Mean age according to different subtypes of NHL NHL: non-Hodgkin lymphoma

NHL Type	Frequency (Percentage)	Mean age ±SD (years)
Diffuse Large B-Cell Lymphoma	179 (71)	53.6±19.4
Burkitt Lymphoma	6 (2.4)	33±15.5
Follicular Lymphoma	34 (13.5)	57.3±14.1
Marginal Zone Lymphoma	10 (4)	71.4±22.3
T cell Non-Hodgkin Lymphoma	15 (6)	39±15.8
Small Lymphocytic Lymphoma	5 (2)	66±11.6
Mantle Cell Lymphoma	3 (1.2)	66.3±6.4
Total	252 (100)	53.9±19.4

**Table 3:** Comparison of prognostic markers among NHL patients ^ Independent sample t test, p<0.05 is considered significant NHL: non-Hodgkin lymphoma

Parameters	Survivor, Frequency (Mean ± SD)	Non-Survivor, Frequency (Mean ± SD)	P-value ^
Age	197 (53.3±19.6)	55 (56.0±18.5)	0.358
Albumin	179 (37.6 ±9.7)	51 (32.9±6.3)	0.001
Lactate Dehydrogenase	193 (227±10)	55 (547±34)	0.000
Hemoglobin	191 (120.6±23)	54 (103±24.7)	0.000

Bone marrow involvement was significantly higher in patients presenting at stage IV, as shown in Table 4. 

**Table 4:** Bone marrow involvement in different NHL stages ^ Chi square test, p<0.05 is considered significant NHL: non-Hodgkin's lymphoma

Stage	Bone Marrow Involvement		Total, n (%)	p value ^
	No, n (%)	Yes, n (%)		
I	42 (91.3%)	4 (8.7%)	46 (100%)	0.000
II	36 (76.6%)	11 (23.4%)	47 (100%)	
III	32 (88.9%)	4 (11.1%)	36 (100%)	
IV	67 (54.5%)	56 (45.5%)	123 (100%)	

DLBCL showed positivity for PAX5 in 108 (60.3%), BCL2 in 106 (59.2%), and MUM1 in 88 (49.2%) cases. Burkitt Lymphoma stained positive for PAX5 in four (66%), c-Myc in three (50%), and EBV in two (33.3%) cases. Of follicular lymphoma cases, 30 (88%) were positive for BCL2, and two (66.7%) mantle cell lymphoma cases were positive for Cyclin D

A total of 197 (78.2%) patients were alive or censored until the last follow-up, whereas 55 (21.8%) did not survive. The mean overall survival was 54.8±39.4 months. Mean survival was higher in older patients compared to younger; however, this was not statistically significant. Subjects in the age group of 20-34 years had a survival of 52.35± 41.3 months, and those in the age group of 35-54 years had a survival of 53.9± 43.3 months. While shortest survival period of 46.4 ±38 months was noticed in the >55 age group.

**Figure 1:**
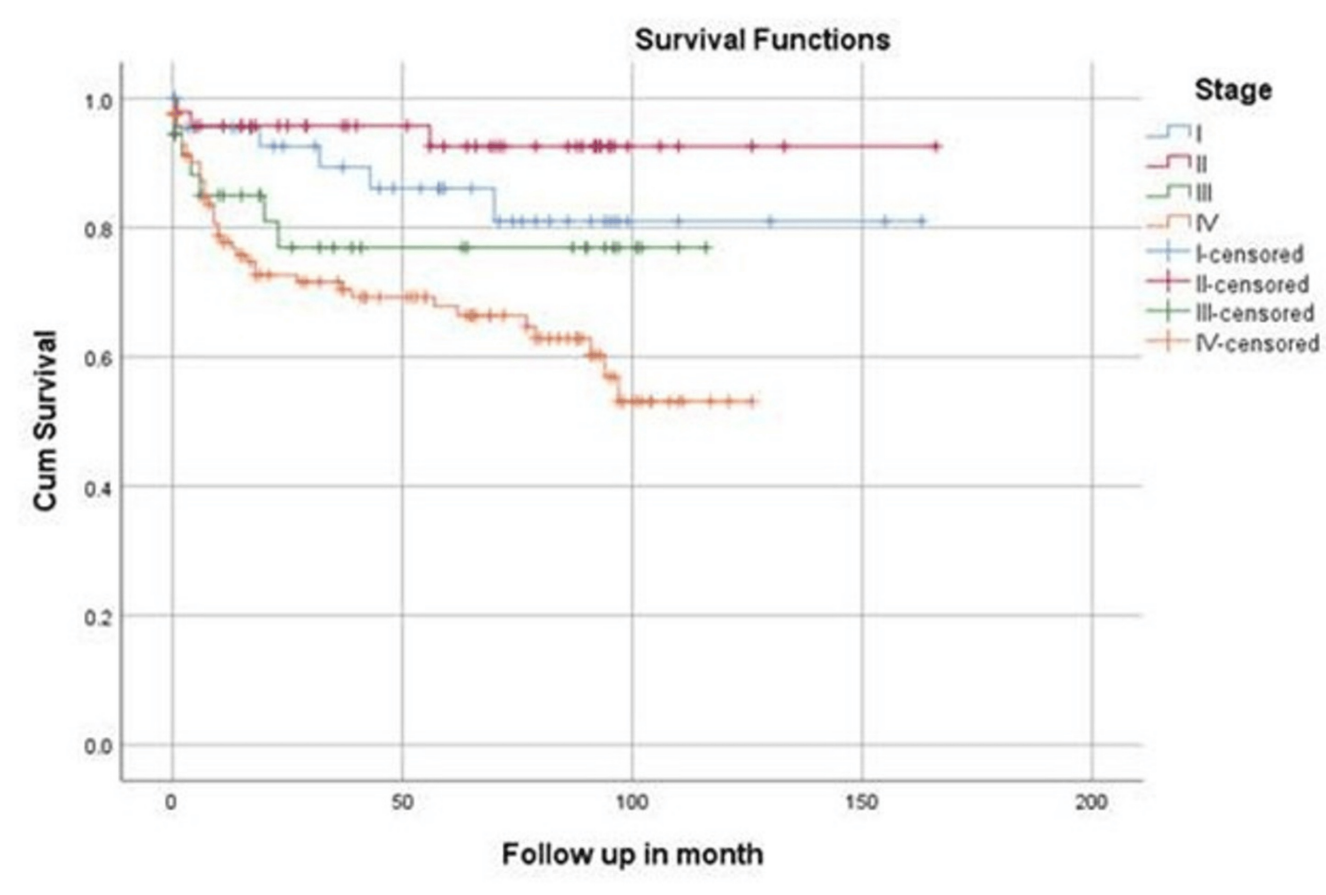
Stage specific survival of NHL patients Kaplan Meier survival curve; Log Rank =17.63, p=0.001 NHL: non-Hodgkin's lymphoma

**Figure 2:**
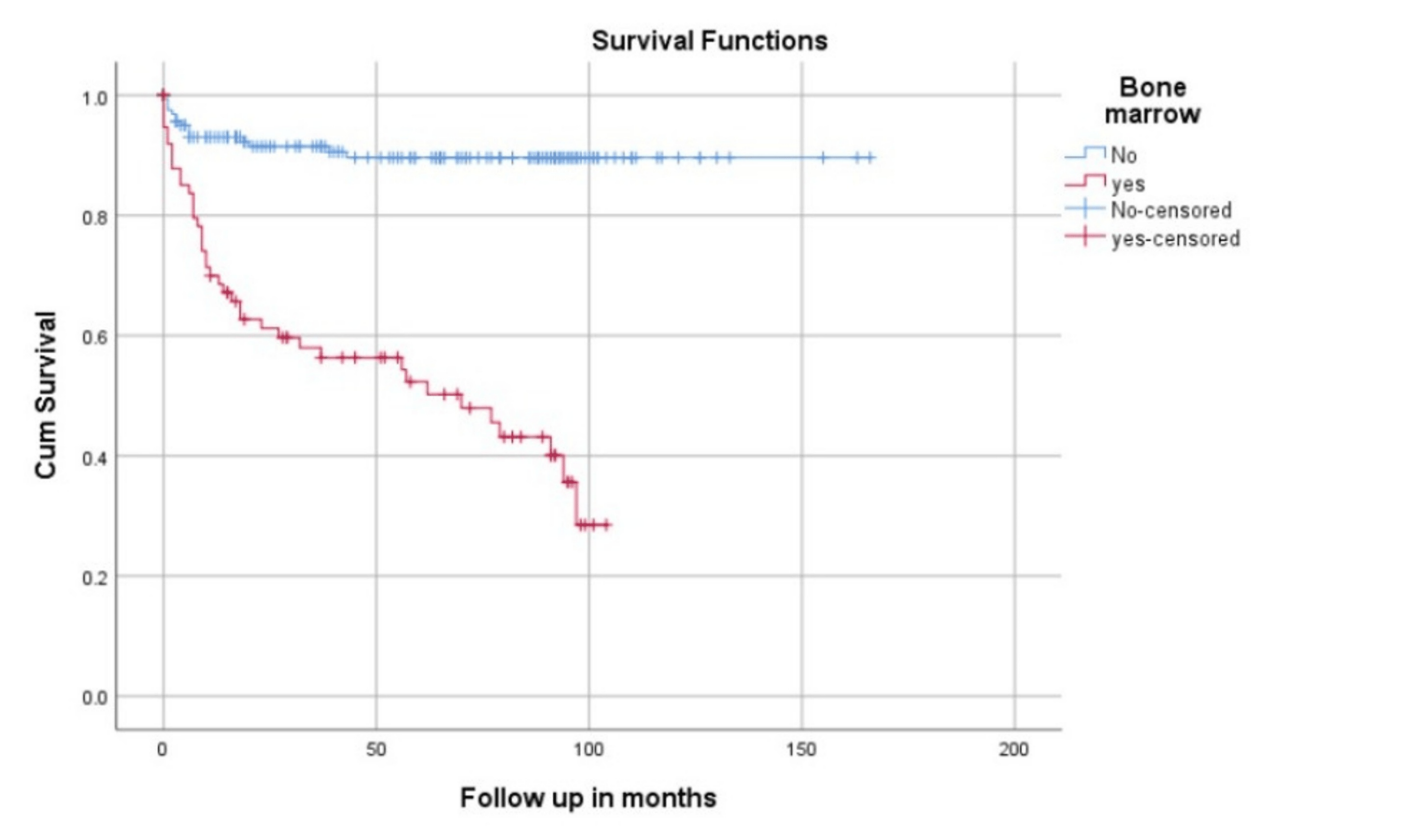
Survival according to bone marrow involvement Kaplan Meier survival curve; Log Rank =56.5, p=0.000

Univariate analysis was done for stage, bone marrow involvement, age, and NHL subtypes using the log-rank test. Advanced stage was associated with poor survival (Log rank 17.7, p=0.001), as displayed in Figure 1. It was also documented that mean survival was significantly higher among patients without bone marrow involvement compared to those with bone marrow involvement (Log rank, 56.5, p=0.000), as illustrated in Figure 2. On univariate analysis no statistically significant difference was observed in the overall survival among the two age groups (Log rank, 1.78, p= 0.4). Similarly, there was no significant association between survival and NHL subtypes (Log rank, 12.2, p=0.05). 

The multivariate Cox proportional hazard model created with age, NHL subtypes, and bone marrow involvement showed that the hazard ratio (HR) for bone marrow involvement was 0.141 (95%CI 0.077-0.258), p≤0.000. Multivariate analysis of stage, albumin, haemoglobin, and LDH level showed that stage IV was associated with poor survival, HR 0.48 (95%CI 0.315-0.742), p=0.001, and low haemoglobin predicted an unfavourable survival with HR 0.978 (95%CI 0.978-0.963), p=0.005.

## Discussion

We studied demographics and prognostic factors of NHL to understand this diverse disease. Our results showed DLBCL as the most common type of NHL, which agrees with previous reports from Saudi Arabia [4,5]. DLBCL makes up approximately 31% of adult NHL cases in the Western population as per the WHO [12]. Similar to our findings, Wang et al. have reported DLBCL to be contributing to one-third of cases of NHL worldwide [14]. This observation reinforces the need for continued innovation in the therapeutic landscape for DLBCL.

Patients in our study presented at a relatively younger age (53.9 years) compared to Western cohorts (67.2 years) [15], while studies conducted in Pakistan have documented a relatively younger age at NHL diagnosis [16,17]. This epidemiological variation points to a distinct disease biology, potentially driven by underlying genetic, environmental, and geographic factors. We observed NHL to be slightly more common among men (58%). A similar trend has been reported by other studies [6-18]. A large proportion of our patients were in stage IV at diagnosis. This finding is consistent with the existing literature from Saudi Arabia [5,19]. This pattern may be driven by several contributing factors, including aggressive clinical course, failure to recognize symptoms, and psychosocial barriers in seeking medical care. 

A low serum albumin was linked to poor survival in our patients. Comparable findings have been reported by other studies [20,21]. A study was carried out on patients undergoing autologous stem cell transplant. Low albumin level before transplantation was an independent risk factor, and these patients needed intensive care [22]. This prompts the need to consider patients with low albumin as a separate cohort needing correction of albumin in addition to the usual treatment of NHL.

LDH levels have shown an inverse relationship with patient survival. Qi et al. conducted a study on DLBCL cases admitted to the intensive care unit. High LDH level was significantly associated with poor prognosis in their patients [23]. Badheeb et al. [24] and Wu et al. [25] found a correlation between elevated LDH levels and reduced survival outcomes in patients with NHL. Cancer cells modify the function of the LDH enzyme to switch to aerobic glycolysis, a phenomenon called the Warburg effect. Such adoption is linked to decreasing oxidative stress and favoring uncontrolled cell division [26].

Our findings revealed that around 78.2% of patients were alive until the last follow-up. This is analogous to research conducted on NHL patients in Riyadh, where 79% of patients were surviving till the final assessment [4]. Our analysis revealed that an advanced disease stage correlated with an unfavorable outcome. Similar association has been reported in other studies, where it was linked to reduced survival rates [11,27,28]. Age was not significantly associated with survival status, which further strengthens the fact that tumor stage and biochemical parameters dictated poor prognosis irrespective of age. Cells progressively acquire genetic mutations, which provide a conducive environment for establishing tumor heterogeneity and metastatic potential. Various genetic, epigenetic alterations, as well as microRNA dysregulations, proteomic and metabolomic signatures kickstart biological processes involved in imparting poor survival in advanced-stage cancers [29].

This study is based on retrospective data, making missing data a limitation. The study population from a tertiary care hospital is likely to be associated with a more aggressive clinical spectrum of disease, which may overestimate the prevalence of advanced clinical stage and hence introduce selection bias. No information was available for a few variables, including microglobulin and performance status, which could have yielded interesting results. The study is underpowered to identify prognostic factors among uncommon histological lymphoma subtypes because of the low numbers of cases. An in-depth multicentre study is required to address less common subtypes.

## Conclusions

The most common NHL subtype was DLBCL. Raised LDH level and decreased albumin level dictated a poor prognosis. Additionally, advanced stage and bone marrow involvement were associated with poor survival. A critical next step is the development and validation of minimally invasive biomarker signatures, including serum-based assays and liquid biopsies, to enable earlier detection of aggressive NHL subtypes in high-risk cohorts.
